# Bafilomycin A1 induces caspase-independent cell death in hepatocellular carcinoma cells via targeting of autophagy and MAPK pathways

**DOI:** 10.1038/srep37052

**Published:** 2016-11-15

**Authors:** Yumei Yan, Ke Jiang, Peng Liu, Xianbin Zhang, Xin Dong, Jingchun Gao, Quentin Liu, Martin P. Barr, Quan Zhang, Xiukun Hou, Songshu Meng, Peng Gong

**Affiliations:** 1 Department of Hepatobiliary Surgery, the First Affiliated Hospital, Dalian Medical University, No. 222 Zhongshan Road, Dalian 116021, China; 2 The First Department of Ultrasound, the First Affiliated Hospital to Dalian Medical University, No. 222 Zhongshan Road, Dalian 116021, China; 3 Institute of Cancer Stem Cell, Dalian Medical University Cancer Center, 9 Lvshun Road South, Dalian 116044, China; 4 Thoracic Oncology Research Group, Trinity Translational Medicine Institute, Trinity Centre for Health Sciences, St. James’s Hospital & Trinity College, Dublin, Ireland; 5 College of Veterinary Medicine, Yangzhou University, Yangzhou, 225009, China; 6 Dalian Key Laboratory of Hepatobiliary Pancreatic Diseases Prevention and Treatment, Dalian 116021, China; 7 Dalian Medical University Graduate School, 9 Lvshun Road South, Dalian 116044, China

## Abstract

Hepatocellular carcinoma (HCC) is refractory to chemotherapies, necessitating novel effective agents. The lysosome inhibitor Bafilomycin A1 (BafA1) at high concentrations displays cytotoxicity in a variety of cancers. Here we show that BafA1 at nanomolar concentrations suppresses HCC cell growth in both 2 dimensional (2D) and 3D cultures. BafA1 induced cell cycle arrest in the G1 phase and triggered Cyclin D1 turnover in HCC cells in a dual-specificity tyrosine phosphorylation-regulated kinase 1B (DYRK1B) dependent manner. Notably, BafA1 induced caspase-independent cell death in HCC cells by impairing autophagy flux as demonstrated by elevated LC3 conversion and p62/SQSTM1 levels. Moreover, genetic ablation of LC3 significantly attenuated BafA1-induced cytotoxicity of HCC cells. We further demonstrate that pharmacological down-regulation or genetic depletion of p38 MAPK decreased BafA1-induced cell death via abolishment of BafA1-induced upregulation of Puma. Notably, knockdown of Puma impaired BafA1-induced HCC cell death, and overexpression of Puma enhanced BafA1-mediated HCC cell death, suggesting a role for Puma in BafA1-mediated cytotoxicity. Interestingly, pharmacological inhibition of JNK with SP600125 enhanced BafA1-mediated cytotoxicity both *in vitro* and in xenografts derived from HCC cells. Taken together, our data suggest that BafA1 may offer potential as an effective therapy for HCC.

Bafilomycin A1 (BafA1), a specific vacuolar H + ATPase (V-ATPase) inhibitor, is frequently used at high concentrations to block the fusion between autophagosomes and lysosomes and as an inhibitor of lysosomal degradation^1^,^2^. Acumulating evidence demonstrates that BafA1 suppresses the growth of a variety of cancer cells^3^,^4^,^5^. In addition to targeting V-ATPase, BafA1 was also found to induce p21-mediated growth inhibition of cancer cells under hypoxic conditions by expressing hypoxia-inducible factor-1alpha (HIF-1α)^6^. Furthermore, BafA1 activated HIF-dependent signaling in human colon cancer cells via mitochondrial uncoupling^7^. Consistent with the effect of BafA1 on autophagy, recent investigations demonstrated that BafA1 targets both autophagy and apoptosis pathways in pediatric B-cell acute lymphoblastic leukemia^8^. In addition, BafA1 has been widely used as an autophagy inhibitor to potentiate the anti-cancer effects of a large number of compounds in pre-clinical trials^9^. These studies suggest that BafA1 may be a promising drug candidate for the treatment of cancer. However, in order to achieve the effective inhibitory effects on cancer cell growth and/or autophagic degradation, BafA1 is usually required at high concentrations (>0.1 μM), which may induce severe acidosis and secondary adverse effects in normal cells, thereby hindering its application in clinical trials.

In this investigation, we show that BafA1 at nanomolar concentrations substantially inhibits the growth of HCC cells in both 2D and 3D cultures and in mouse models. We further demonstrate that BafA1 induces caspase-independent HCC cell death via targeting of autophagy and MAPK pathways. Our data supports further exploration of BafA1 as a drug candidate in the treatment of HCC.

## Results

### BafA1 inhibits human HCC cell growth, colony & spheroid formation and lyses spheroids

**Figure 1:**
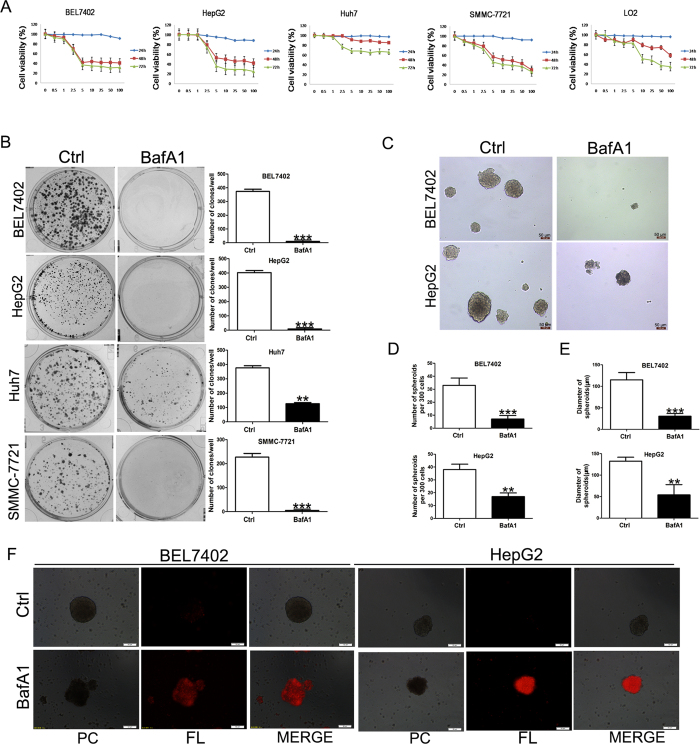
Bafilomycin A1 inhibits human HCC cell growth and spheroid formation. (**A**) Hepatocellular carcinoma cell lines (BEL7402, HepG2, Huh7 and SMMC-7721) and LO2 cells were vehicle-treated or treated with varying concentrations of BafA1 (0.5, 1, 2.5, 5, 10, 25, 50 and 100 nM) for 24, 48, 72 h. Cell growth inhibition was determined using the MTT assay. (**B**) Hepatocellular carcinoma cell lines (BEL7402, HepG2, Huh7 and SMMC-7721) were vehicle-treated or treated with 5 nM BafA1 and cultured in complete media for 14 days for colony formation analysis. (**C**) BEL7402 and HepG2 cells were pre-treated as in (**B**) for 24 h and seeded in ultra-low attachment 96-well plates for 7 days. Scale bar = 50 μm. (**D**) BEL7402 and HepG2 cells were seeded in ultra-low attachment 96-well plates for 7 days, and treated the same as in (**B**). The number of spheroids/300 cells was quantified. (**E**) BEL7402 and HepG2 cells were seeded in ultra-low attachment 96-well plates for 7 days, and treated the same as in (**B**). The diameter of spheroids was quantified. (**F**) Following treatment for 24 h, spheroids were stained with PI and imaged under phase contrast (PC) and red fluorescence microscopy (FL) (scale bar = 50 μm). Data are presented as Mean ± SEM from three independent experiments, (BafA1 means Bafilomycin A1).

To investigate the effect of BafA1 on HCC cells, BEL7402, HepG2, Huh7 and SMMC-7721 cells were treated with BafA1 and a number of cell-based analyses were performed. The non-transformed human liver cell line, LO2 was also included. MTT assays were carried out to determine the growth kinetics of the cell lines in response to BafA1 at increasing concentrations for 24–72 h. As shown in Fig. 1A, treatment with 5 nM BafA1 for 48 or 72 h significantly inhibited the growth of BEL7402, HepG2, Huh7 and SMMC-7721 cells, but had no effect on LO2 cells. However, treatment with 10 nM BafA1 for 72 h resulted in approximately a 50% reduction in growth of LO2 cells (Fig. 1A). As such, BafA1 at 5 nM was used in subsequent experiments. Treatment with 5 nM BafA1 for 14 days robustly inhibited the colony formation ability of HCC cells (Fig. 1B). Moreover, BafA1-treated BEL7402 and HepG2 cells did not form spheroids under 3D culture conditions compared to control cells treated with 0.05% DMSO (Fig. 1C). This would suggest an inhibitory effect of BafA1 in abrogating the 3D growth potential of HCC cells. Furthermore, both the number and volume of BEL7402 and HepG2 spheroids, when treated with BafA1 for 7 days, were significantly reduced over time (Fig. 1D,E). Notably, large amounts of the cells from BafA1-treated BEL7402 and HepG2 spheroids were stained with the cell-death dye propidium iodide (PI), indicative of cell death (Fig. 1F). Together, these results indicate that BafA1, at low concentration (5 nM), is sufficient to inhibit HCC cell growth *in vitro*.

### BafA1 induces HCC cell cycle arrest and DYRK1B-dependent Cyclin D1 turnover

**Figure 2:**
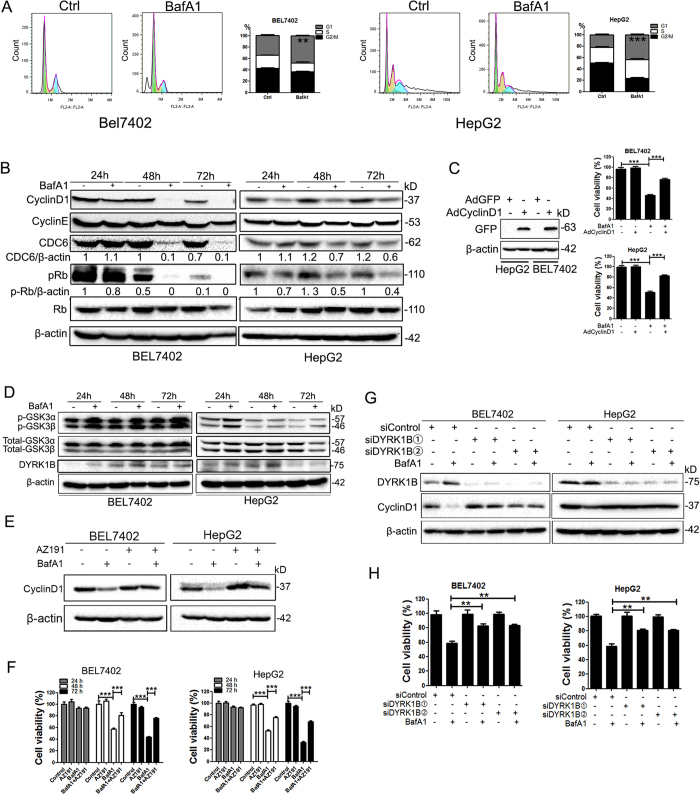
Bafilomycin A1 induces HCC cell cycle arrest and DYRK1B-dependent Cyclin D1 turnover. (**A**) BEL7402 and HepG2 cells were vehicle-treated or treated with 5 nM BafA1 for 24 h, stained with propidium iodide and cell cycle analyzed by FACS. (**B–D**) BEL7402 and HepG2 cells were treated the same as in (**A**) for 24, 48, 72 h. (**B**) Protein expression of Cyclin D1, Cyclin E, CDC6, pRb and Rb were analyzed by IB. (**C**) BEL7402 and HepG2 cells were infected with adenoviruses expressing GFP-Cyclin D1 or vector control at a multiplicity of infection of 250. Cells were treated with BafA1 and cell viability determined using the MTT assay. (**D**) Protein expression of p-GSK3α/β, total GSK3α/β, DYRK1B was analyzed by Immunoblotting in response to treatment with BafA1 over 72 h. (**E**) BEL7402 and HepG2 cells were treated with vehicle or 10 μM of the DYRK1B inhibitor, AZ191, alone and in combination with BafA1 for 24 h. Cyclin D1 expression was examined by immunoblot analysis. (**F**) BEL7402 and HepG2 cells were treated as in (**E**) for 24, 48 and 72 h, and determined by MTT for cell viability analysis. (**G**) BEL7402 and HepG2 cells were transfected with two siDYRK1B oligonucleotides or a negative control (siControl) for 48 h, followed by treatment with BafA1. Immunoblot analysis was used to examine the expression of DYRK1B and Cyclin D1. For all immunoblots, β-actin was used as a control for equal loading. (**H**) BEL7402 and HepG2 cells were transfected as in (**G**), then treated with BafA1 and cell viability was measured using the MTT assay. Data are presented as mean ± SEM from three independent experiments, (BafA1 means Bafilomycin A1).

To dissect the mechanism by which BafA1 suppresses HCC cell growth, we first examined whether BafA1 perturbs the cell cycle distribution. BafA1 (5 nM) increased the percentage of BEL7402 and HepG2 cells in the G1 phase of the cell cycle, with a concomitant decrease in cells in G2/M phases, indicative of a G1 phase arrest (Fig. 2A). Consistently, exposure to BafA1 induced a time-dependent reduction in G1 phase regulatory proteins such as Cyclin D1, CDC6 (cell division cycle 6) and pRb in BEL7402 and HepG2 cells (Fig. 2B). Notably, adenovirus-mediated overexpression of Cyclin D1 impaired BafA1-mediated inhibitory effects on the growth of HCC cells (Fig. 2C), suggesting a critical role of Cyclin D1 in BafA1 activity in HCC cells.

Cyclin D1 levels in mammalian cells is mainly controlled by glycogen synthase kinase 3β (GSK3β)^10^. However, activation of GSK3β (dephosphorylation level) was not observed in BafA1-treated HCC cells compared with mock-treated cells (Fig. 2D), suggesting that GSK3β may not be implicated in BafA1-induced Cyclin D1 turnover in HCC cells. Previous studies, in addition to more recent work in our laboratory have shown that DYRK1B (dual-specificity tyrosine phosphorylation-regulated kinase 1B) promotes the turnover of Cyclin D1 in a GSK3β-independent manner^11^,^12^. A steady-state increase in DYRK1B protein levels was observed in both BEL7402 and HepG2 cells following treatment with BafA1 (Fig. 2D). Moreover, AZ191, a selective inhibitor of DYRK1B^11^, reversed Cyclin D1 turnover induced by BafA1 in both BEL7402 and HepG2 cells (Fig. 2E), suggesting that a role for DYRK1B in BafA1-triggered turnover of Cyclin D1 in HCC cells. Consistently, AZ191 treatment decreased BafA1-induced e inhibitory effect on HCC cell growth (Fig. 2F). Supporting this notion, siRNA-mediated knockdown of DYRK1B in either BEL7402 or HepG2 cells decreased the effect of BafA1 on Cyclin D1 degradation and HCC cell growth (Fig. 2G,H). These data indicates that DYRK1B regulated BafA1-mediated cell growth inhibition of HCC cells by regulating Cyclin D1.

### BafA1 triggers caspase-independent cell death in HCC cells

**Figure 3:**
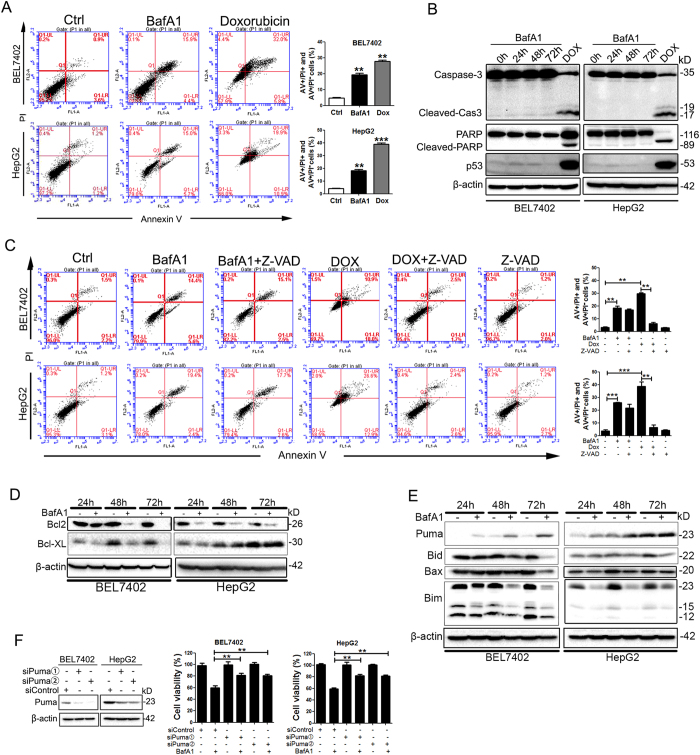
BafA1 triggers caspase-independent cell death. BEL7402 and HepG2 cells were treated with vehicle control, 5 nM BafA1 or using 2 μM Doxorubicin (Dox) as a positive control. (**A**) After 48 h, cells were stained using Annexin V/PI double-staining and analyzed by FACS. (**B**) At 24, 48 and 72 h, Caspase-3, PARP and p53 expression was examined by IB, using β-actin as a loading control. (**C**) Cells were treated with BafA1 in the presence or absence of the caspase inhibitor, Z-VAD-FMK (50 μM) for 48 h double stained with Annexin V/PI and analyzed by FACS. (**D,E**) BEL7402 and HepG2 cells were treated for 24, 48 and 72 h using BafA1 and expression of Bcl2, Bcl-XL, Puma, Bid, Bax and Bim was examined by IB. (**F**) BEL7402 and HepG2 cells were transfected with two siPuma or siControl oligonucleotides for 48 h, treated with BafA1 for 24 h and cell viability was measured using the MTT assay. Data are presented as mean ± SEM from three independent experiments, (BafA1 means Bafilomycin A1).

To determine whether BafA1-induced growth inhibition in HCC cells was due to cell death, BafA1-treated BEL7402 and HepG2 cells were analyzed by flow cytometry with FITC-conjugated Annexin-V and PI double staining. As illustrated in Fig. 3A, BafA1 treatment increased the percentage of both early and late apoptotic cells in both BEL7402 and HepG2 cell lines, suggesting BafA1-mediated induction of apoptotic cell death. Doxorubicin (Dox, 2 μM) was used a positive control, which significantly increased the number of apoptotic cells (Fig. 3A). However, two classical apoptosis markers, caspase-3 activation and Poly (ADP-ribose) polymerase (PARP) cleavage, were not observed in BafA1-treated BEL7402 or HepG2 cells (Fig. 3B). By contrast, Dox treatment induced marked cleavage of caspase-3 and PARP in addition to increased p53 levels (Fig. 3B). Furthermore, pre-treatment with a broad-specificity caspase inhibitor Z-VAD-FMK had no obvious effect on BafA1-induced cell death in BEL7402 and HepG2 cells, whereas Dox-induced cell death was inhibited by pre-exposure to Z-VAD-FMK (Fig. 3C). These data suggest that BafA1 may not induce caspase-dependent cell death in HCC cells. We also examined the expression levels of the Bcl2 family of proteins, which are reportedly involved in BafA1-induced cancer cell death^8^. While BafA1 treatment downregulated the levels of pro-survival proteins Bcl2 and Bcl-XL in HCC cells (Fig. 3D), the expression levels of several pro-apoptotic proteins such as Bax, Bid and Bim were also downregulated (Fig. 3E). We also observed that Puma expression were upregulated in BEL7402 and HepG2 cells following BafA1 treatment for 24 h. Interestingly, siRNA-mediated knockdown of Puma in BEL7402 and HepG2 cells significantly inhibited the cytotoxicity exerted by BafA1 (Fig. 3F), indicating a role of Puma in BafA1-mediated cytotoxicity of HCC cells. In addition, BafA1 did not induce the expression of RIP1 or RIP3, two key mediators of necrosis, in BEL7402 and HepG2 cells (data not shown).

### BafA1 inhibits autophagy fluxs

**Figure 4:**
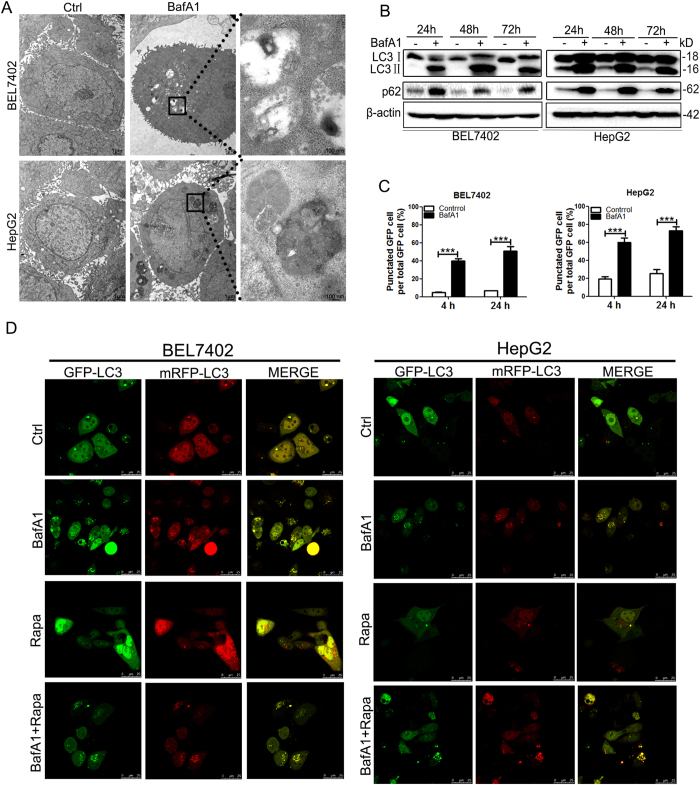
BafA1 inhibits autophagy flux in HCC cells. (**A**) BEL7402 and HepG2 cells were treated with vehicle control or with 5 nM BafA1 for 24 h, and analyzed by transmission electron microscopy (TEM). Typical structures enclosed in the black square have been enlarged in the right panel for more detailed imaging. (**B**) Cells were also treated as above (**A**), and protein expression was examined using IB analysis for LC3, p62 and β-actin. (**C**) Following transfection of BEL7402 and HepG2 cells with GFP-LC3 for 24 h, cells were treated for 4 and 24 h. The numbers of cells with GFP-LC3 punctate are represented. (**D**) BEL7402 and HepG2 cells were treated for 24 h following transfection with GFP-mRFP-LC3. Images were captured by confocal microscopy (60 × oil immersion lens), scale bar = 25 μm. Data are presented as mean ± SEM from three independent experiments, (BafA1 means Bafilomycin A1).

Recent studies indicate that BafA1 triggers caspase-independent cell death in cancers of different origins^8^,^13^. Given the established role of BafA1 in blocking autophagosome-lysosome fusion during the late stage of autophagy, we hypothesized that BafA1 may target autophagy to induce cell death of HCC cells. Transmission electron microscopy (TEM)-based analysis revealed increased formation of double-membrane vesicles (autophagosomes) in BafA1-treated BEL7402 or HepG2 cells but not in vehicle-treated cells (Fig. 4A). The increased autophagosomes may be due to either autophagy induction or inhibition of autophagic flux. Degradation of p62 is generally used as an autophagy flux marker^14^. Exposure of cells to BafA1 over time induced robust LC3 conversion (i.e. cytosolic LC3I to autophagosome-bound lipidated LC3II) and increased p62 levels in both BEL7402 and HepG2 cells (Fig. 4B), thereby suggesting that BafA1 blocks autophagy flux in HCC cells.

To confirm that the observed double-membrane vesicles were indeed related to autophagy, GFP-LC3 dot formation was investigated. HCC cells were transfected with GFP-LC3. The number of cells with GFP-LC3 punctate in BafA1-treated cells was significantly higher than in control cells (Fig. 4C). The effect of BafA1 on autophagy flux was further investigated using a tandem-tagged GFP-mRFP-LC3 plasmid, which is based on the finding that GFP, but not mRFP is quenched upon autophagic delivery of this protein to the acidic environment of the lysosome^2^. Therefore, autophagosomes display both green and red fluorescence, while autolysosomes appear only red. Numbers of red-only (acidic) and red plus green (yellow, non-acidic) autophagic vesicles were assessed in HCC cells transfected with GFP-mRFP-LC3 plasmid subjected to BafA1 treatment. BafA1 (5 nM) treatments induced a significant increase in the number of yellow autophagosomes in either BEL7402 or HepG2 cells compared with control cells (Fig. 4D). As expected, treatment with rapamycin (Rapa), a known autophagy inducer^2^, significantly increased the number of red autolysosomes in HCC cells. Together, these results indicate that BafA1, at low concentrations, blocks autophagosome-lysosome fusion in HCC cells.

### Inhibition of autophagosome formation decreases BafA1-mediated cell death

**Figure 5:**
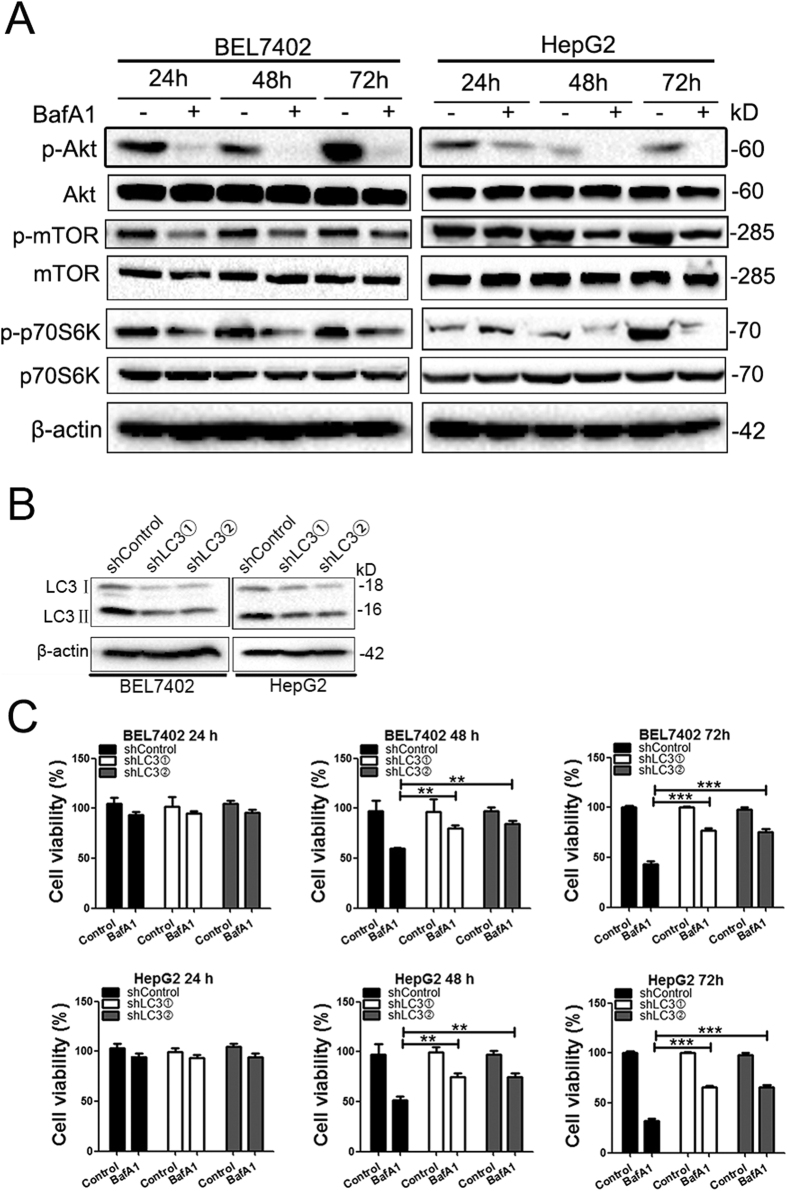
Inhibition of autophagy induction decreases BafA1-mediated cell death. (**A**) BEL7402 and HepG2 cells were vehicle-treated or treated with 5 nM BafA1 for 24, 48, 72 h. Protein expression of p-Akt, Akt, p-mTOR, mTOR, p-p70S6K and p70S6K were analyzed by IB, using β-actin as a loading control. (**B**) Cell lysates reprsenting BEL7402/shLC3, HepG2/shLC3 or BEL7402/shControl and HepG2/shControl cells were analyzed by IB to determine the efficiency of LC3 knockdown. (**C**) BEL7402/shLC3, HepG2/shLC3 or control cells (BEL7402/shControl and HepG2/shControl) were treated with vehicle control or 5 nM BafA1 for 24, 48 and 72 h, after which time cell viability was determined using the MTT assay. Data are presented as mean ± SEM from three independent experiments, (BafA1 means Bafilomycin A1).

We next examined the role of autophagy in BafA1-induced HCC cell death. A recent report by Yuan *et al.* showed that BafA1 at low concentration (1 nM) inhibits the early stage of autophagy by promoting mTOR activation and Beclin1 association with Bcl2 in pediatric B-cell ALL cells^8^. We investigate whether BafA1 at low concentration inhibits the the early stage of autophagy by promoting mTOR activation in HCC cell lines as observed in pediatric B-cell ALL cells. Surprisingly, we observed that BafA1 treatment (5 nM) reduced the phosphorylation levels of Akt, mTOR and p70S6K in both Bel7402 and HepG2 cells in a time-dependent manner (Fig. 5A). No change was detected in the levels of total Akt, mTOR, and p70S6K. These observations indicated that BafA1 treatment (5 nM) inhibits the activation of the class I PI3K/Akt/mTOR/p70S6K signaling pathway in HCC cell lines, suggesting a cell or cancer-type specific effect by BafA1 at low concentration. Given that the class I PI3K/Akt/mTOR/p70S6K signaling pathway negatively regulates autophagy induction, BafA1 treatment at low concentration may result in autophagy induction (autophagosome formation) in HCC cells.

We thus examine whether autophagosome formation is involved in BafA1-mediated inhibitory effects on HCC cell growth. To this end, we established BEL7402 and HepG2 cells in which LC3 was stably depleted with lentiviruses carrying specific shRNA. The efficiency of knockdown was confirmed by immunoblot assay (Fig. 5B). As shown in Fig. 5C, depletion of LC3 significantly attenuated BafA1-induced growth inhibition of HCC cells.

### Down-regulation of p38 MAPK or JNK pathways alters BafA1-induced cell death

**Figure 6:**
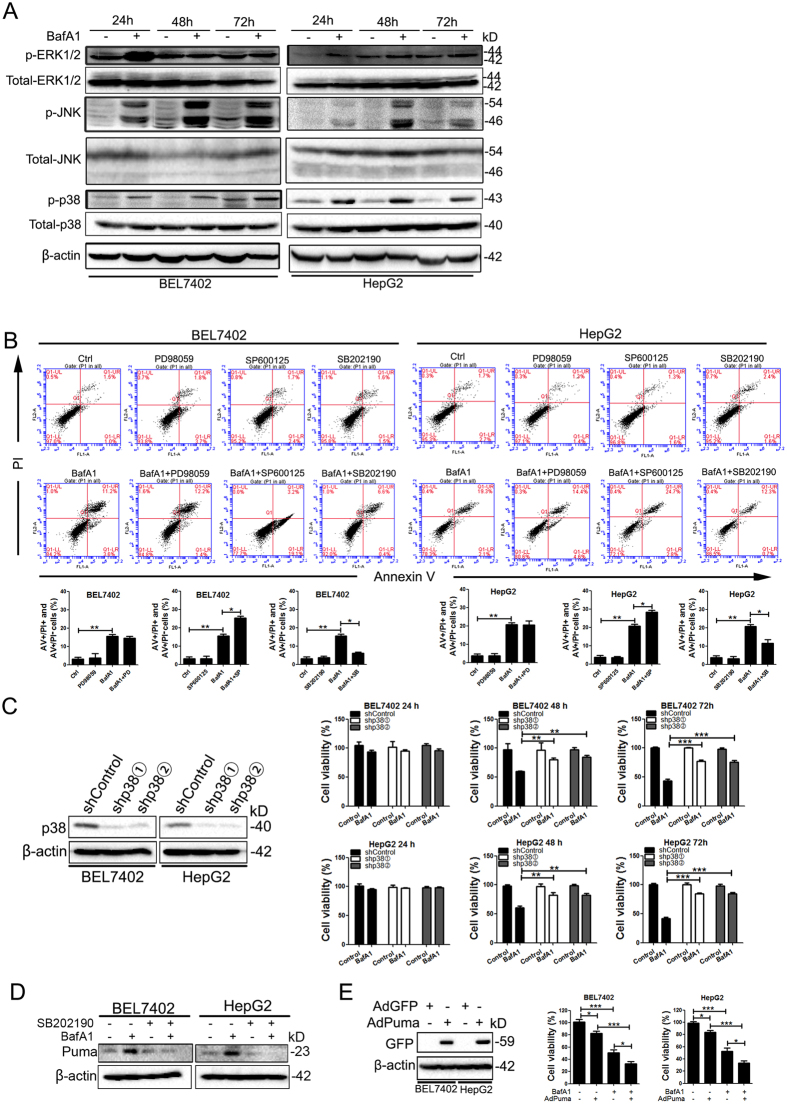
Down-regulation of the p38 MAPK pathway decreases BafA1-induced cell death. (**A**) BEL7402 and HepG2 cells were treated with vehicle controls or with 5 nM BafA1 for 24, 48 and 72 h. IB analysis for p-ERK1/2, p-JNK, p-p38, total-ERK1/2, total-JNK, and total-p38 was used to examine protein expression, using β-actin as a loading control. (**B**) HCC cells were pre-treated with inhibitors to either ERK (PD98059), JNK (SP600125), or p38 (SB202190) and subsequently treated with BafA1 alone, or in combination, for 24 h, double stained with Annexin V/PI and analyzed by FACS. (**C**) HCC cells were transfected with a stable knockdown for p38 (BEL7402/shp38 and HepG2/shp38), or control cells (BEL7402/shControl and HepG2/shControl) and were treated with vehicle control or 5 nM BafA1 for 24, 48, and 72 h. At each time-point, cell viability was examined using the MTT assay. (**D**) BEL7402 and HepG2 cells were pre-treated SB202190, vehicle control or 5 nM BafA1 for 24 h. IB analysis for protein expression of Puma was carried out, using β-actin as a loading control. (**E**) BEL7402 and HepG2 cells were infected with adenoviruses expressing GFP-Puma or vector control at a multiplicity of infection of 250. Cells were treated with BafA1 and cell viability determined using the MTT assay. Data are presented as mean ± SEM from three independent experiments, (BafA1 means Bafilomycin A1).

The three major mitogen-activated protein kinase (MAPK) pathways (ERK, JNK and p38) are known regulators of cell survival, proliferation and stress response. Consistent with previous observations in breast cancer cells^13^, phosphorylation levels of ERK, JNK and p38 were increased in response to BafA1 treatment in HCC cells (Fig. 6A). To investigate whether MAPK activity alters BafA1-induced cell death, selective inhibitors of each of these pathways were used and cell death was quantified by flow cytometry analysis. Inactivation of these targeted pathways was also confirmed by immunoblot analysis (Fig. S1). Inhibition of MEK by PD98059 did not display a significant effect on BafA1-induced HCC cell death (Fig. 6B). However, pre-treatment of BEL7402 and HepG2 cells with the JNK inhibitor, SP600125, significantly enhanced BafA1-induced cell death (Fig. 6B), suggesting that a combination of BafA1 with a JNK inhibitor may be a potential strategy to enhance the effects of BafA1 in mediating HCC cell death. On the other hand, the p38 MAPK inhibitor, SB202190, significantly decreased BafA1-induced cell death (Fig. 6B). Consistently, genetic ablation of p38α with lentivirus-mediated shRNA in BEL7402 and HepG2 cells significantly abrogated this BafA1-mediated effect on HCC cell death (Fig. 6C). These data suggest that the p38 MAPK pathway may be required for the full effect of BafA1 on HCC cell death.

To dissect the underlying mechanism of action of p38 MAPK in BafA1-induced cytotoxicity of HCC cells, protein expression of LC3II and p62 in addition to several members of the Bcl2 family of proteins were determined by immunoblot analysis. Both LC3II conversion and p62 accumulation by BafA1 in BEL7402 and HepG2 cells were decreased following pre-treatment with SB202190 (Fig. S1C), indicating that inhibition of p38 MAPK activation impaired BafA1-inhibited autophagy flux. On the other hand, SB202190 substantially abolished BafA1-induced upregulation of Puma in HCC cells (Fig. 6D). In addition, adenovirus-mediated overexpression of Puma enhanced BafA1-mediated inhibitory effects on the growth of HCC cells (Fig. 6E), indicating that p38 regulated BafA1-mediated cytotoxicity of HCC cells by Puma.

### BafA1 suppresses HCC tumor growth in a xenograft model, an effect that is enhanced by JNK inhibition

**Figure 7:**
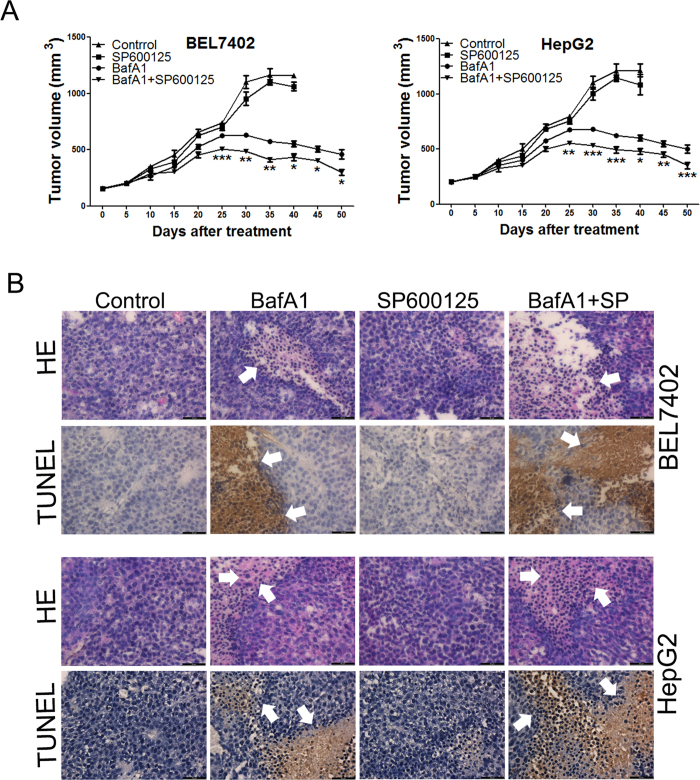
BafA1 suppresses HCC growth in mouse tumor xenografts and is enhanced by JNK inhibition. Six week old female nude mice bearing BEL7402 and HepG2 tumors were randomized into four groups: (a) vehicle-treated, (b) SP600125 (intraperitoneal (ip) three times per week), (c) BafA1 (10 mg/kg) via intratumoral administration three times per week, (d) BafA1 treatment in combination with SP600125, the inhibitor (SP600125) was administered one day prior to treatment with BafA1. (**A**) Tumor volumes were measured at 5-day intervals for 50 days and expressed as the mean ± SD (n = 10) in tumor volume-time curves. Differences in tumor regression were significant between BafA1-treated mice and vehicle control groups and in mice receiving combined treatments and treatment alone (BafA1 or drugs alone) (*p < 0.05; **p < 0.01; ***P < 0.001). No statistical significance was observed between mice receivsing single treatments alone and vehicle controls (**B**) Five weeks after treatment, tumor tissue samples from each treatments group were subjected to either hematoxylin-eosin (H&E) staining (the upper panels, tumor necrosis indicated by white arrows) or TUNEL assay (the lower panels, arrowheads indicate brown 3,3′-diaminobenzidine chromogen in the cell nuclei of apoptotic cells), (BafA1 means Bafilomycin A1).

We further examined the effect of BafA1 on HCC growth in xenografts derived from BEL7402 and HepG2 cells. BafA1 markedly abrogated the progression of established tumors compared with controls (Fig. 7A, p < 0.05). Notably, combination with the JNK inhibitor, SP600125, further enhanced this BafA1-mediated decrease in tumor size (Fig. 7A). Further histologic examination of harvested tumors showed increased inflammatory cell infiltration and cell death in tumors treated with BafA1 alone, or combination with SP600125 (Fig. 7B). No weight loss was observed in control mice injected with BafA1, indicating that this treatment was well tolerated (data not shown).

## Discussion

High concentrations of BafA1 (>0.1 μM) have been widely used in the inhibition of lysosomal activity, blocking autophagy flux and/or inducing cytotoxicity in various cancer types, including HCC^3^. However, BafA1 at high concentration may also exert marked cytotoxicity in normal cells, thus hindering its specificity and use in clinical studies. In line with a recent report showing that 1 nM BafA1 effectively and specifically inhibited the growth of pediatric B-cell acute lymphoblastic leukemia cells^8^, in this study we provide evidence that 5 nM BafA1 significantly inhibited HCC cell growth *in vitro* and 10 mg/kg BafA1 markedly abrogated the progression of established tumors. Furthermore, the *in vivo* effect of BafA1 on HCC cells was enhanced in response to combination treatment with BafA1 and the JNK inhibitor, SP600125. Given that HCC is refractory to almost all forms of chemotherapy, our data suggest that BafA1 alone, or in combination with a JNK inhibitor, may represent a promising therapeutic approach in HCC.

Several studies have documented that BafA1 induces cell cycle arrest in cancer cells, a process involving deregulation of cell cycle regulators^5^,^8^. However, the molecular mechanism by which BafA1 exerts its effect on these cell cycle regulators remains poorly understood. Here, we show that pharmacological inhibition or genetic depletion of DYRK1B, but not GSK3β, reversed cyclin D1 turnover induced by BafA1 in HCC cells, suggesting a critical role of DYRK1B in BafA1-induced cell cycle arrest. It is known that DYRK1B promotes turnover of cyclin D1 in a GSK3β-independent manner^11^, supported by our recent observation that DYRK1B plays a role in LGX818-induced cyclin D1 turnover, a third generation BRAF inhibitor^12^. In this investigation, we further show that inhibition of DYRK1B reduced BafA1-induced HCC cell death, supporting its role as a mediator of the anti-cancer effects of BafA1.

BafA1 displays anti-cancer effects in a variety of cancers including HCC via diverse mechanisms such as autophagy and/or apoptosis^3^,^6^,^15^,^16^,^17^. For instance, BafA1 at low concentration induces caspase-independent cell death in pediatric B-cell acute lymphoblastic leukemia cells via targeting of autophagy and apoptosis^8^. The authors further showed that BafA1 inhibits the early stage of autophagy by promoting mTOR activation and Beclin1 association with Bcl2 in pediatric B-cell ALL cells^8^. In the current study, we observed that BafA1(5 nM) triggers caspase-independent cell death in HCC cells, where no caspase activation and PARP cleavage was observed. However, marked LC3 conversion and accumulation of p62 was detected in BafA1-treated HCC cells, indicating that BafA1 impairs the autophagy flux. Inconsistent to the findings by Yuan *et al.* in BafA1- treated pediatric B-cell ALL cells, we observed that BafA1 treatment inhibits the activation of the class I PI3K/Akt/mTOR/p70S6K signaling pathway, suggesting that BafA1 treatment at low concentration may result in autophagy induction in HCC cells. Therefore, in BafA1-treated HCC cells, the observed LC3II accumulation (Fig. 4B), the increased number of cells with GFP-LC3 punctate (Fig. 4C) may be partially caused by BafA1-induced early stage of autophagy. Given that BafA1 inhibited autophagosome-lysosome fusion in HCC cells as shown by the accumulation of autophagic flux indicator p62 (Fig. 4B) and yellow autophagosomes (Fig. 4D), we proposed that BafA1 may induce early stage of autophagy (via inhibiting the Akt/mTOR/p70s6k pathway) and inhibit later stage of autophagy (autophagosome-lysosome fusion) in HCC cell lines. In this way, more autophagic vacuoles may be accumulated than inhibiting later stage of autophagy only. Thus, konckdown of LC3 in HCC cells will blunt BafA1-induced early stage of autophagy. Consistently, depletion of LC3 significantly attenuated BafA1-induced growth inhibition of Bel7402 and HepG2 cells, suggesting that BafA1 induces HCC cell death via targeting of autophagy.

In addition to targeting autophagy, BafA1 up-regulated the three major MAPK pathways (ERK, JNK, p38) in HCC cells, consistent with previous observations in breast cancer cells^13^. Furthermore, we show that p38, but not ERK or JNK, was required for BafA1- induced HCC cell death as pharmacological inhibition or genetic depletion of p38 reduced the cytotoxicity of BafA1. One possible mechanism of action of p38 in this BafA1-mediated effect is via the regulation of Puma, which was upregulated by BafA1 and downregulated in response to treatment with a p38 inhibitor or as a result of its knockdown. Other mechanisms may also be associated with p38-mediated regulation of autophagy as p38 inhibition reduced BafA1-induced LC3 conversion and p62 accumulation. Intriguingly, pharmacological inhibition of JNK enhanced BafA1-induced HCC cell death both *in vitro* and in mouse tumor xenografts. Taken together, our results strongly suggest that multiple signaling pathways, including those involved in autophagy and MAPK pathways play a role in BafA1-induced HCC cell death. Our results also reveal that Puma plays a critical role in BafA1-induced HCC cell death as genetic depletion of Puma hindered the inhibitory effect of BafA1, highlighting Puma as a potential target for BafA1-mediated cell death in HCC.

With the increasing incidence of HCC in the Europea and Asia in recent years^18^, we conclude that BafA1 may be a promising therapeutic target in human HCC and as such, warrants further studies.

## Methods

### Cell lines

BEL7402, LO2 and SMMC-7721 cells were maintained in RPMI 1640 cell culture media. Huh7 cells were cultured in DMEM and HepG2 cells were maintained in EMEM. All culture media were supplemented with 10% fetal bovine serum (FBS) and 1% penicillin/streptomycin (PS). Cells were cultured in a humidified incubator in 5% CO_2_ at 37 °C.

### Antibodies and reagents

Anti-microtubule-associated protein 1 light chain 3 (LC3), p62 and β-actin were obtained from Sigma. Anti-Cyclin D1, Cyclin E, CDC6 and Rb were obtained from Santa Cruz. The following, antibodies were purchased from Cell Signaling Technology: phospho-ERK1/2, phospho-p38, phospho-JNK, phospho-GSK3α/β, phospho-Rb, p53, Caspase-3, Bcl2, Puma, Beclin-1 and DYRK1B, along with antibodies directed against Erk1/2, p38, JNK and GSK3α/β. The inhibitors PD98059, SB202190, SP600125 and AZ191 were purchased from Selleck. Bafilomycin A1 (BafA1) was purchased from Merck Millipore. Drugs were dissolved in dimethyl sulfoxide (DMSO) as stock solutions and stored at −20 °C.

### Lentiviral constructs and stable cell lines

The following lentiviral constructs were purchased from Santa Cruz: MAP LC3β shRNA (sc-43390-V), p38α shRNA (sc-29433-V) and noncoding shRNA (sc-108080). Lentiviral particles were used to directly infect BEL7402 and HepG2 cells. Stable clones were subsequently selected using puromycin (Sigma).

### RNA interference

RNA interference was used to knock down DYRK1B and Puma. DYRK1B①: 5′-GGUGAAAGCCUAUGAUCAUTT-3′; DYRK1B②; 5′-GCCUGGUAUUUGAGCUGCUGUCCUA-3′. Puma①: 5′-GCCUGUAAGAUACUGUAUATT-3′; Puma②: 5′-GGAGGGUCCUGUACAAUCUTT-3′. A scrambled siRNA was used as a negative control: 5′-UUCUCCGAACGUGUCACGUTT-3′.

### Cell proliferation and colony formation assays

Tumor cell growth was measured daily using the MTT (3-(4,5-dimethyl-2-thiazolyl)-2,5-diphenyl tetrazolium bromide) assay as previously described^12^. To determine colony formation, hepatoma carcinoma cells were cultured in complete medium for 14 days. Colonies (containing 50 or more cells) were counted by light microscopy.

### Spheroid formation

BEL7402 and HepG2 cells were seeded (300 cells/well) in ultra-low attachment 96-well plates and maintained in serum-free DMEM/F12 medium supplemented with 10 ng/ml basic fibroblast growth factor (bFGF), 20 ng/ml epidermal growth factor (EGF) and B27 (B27 and medium at a 1:50 volume ratio). Seven days after seeding, the propagated spheroid bodies were collected and digested by StemPro Accutase to single cell suspensions to generate a second generation of spheroids^19^.

### Immunoblotting

Cells were treated with various agents as indicated in figure legends. After treatment, cells were collected and processed for immunoblotting analysis as previously described^20^. To quantify changes, the densitometries of protein bands were determined with a calibrated GS-670 densitometer.

### Cell cycle and apoptosis

For cell cycle analyses, cells were treated with vehicle or BafA1 for 24 h and then were collected and fixed in cold 70% ethanol overnight at 4 °C. To ensure that only DNA was stained, cells were treated with PBS (contain 100 μg/mL RNase A, 50 μg/mL PI and 0.2% Triton X-100) and then were incubated for 10 min at room temperature in the dark. All samples were analyzed by flow cytometry. For analysis of apoptosis, cells were treated with vehicle or BafA1 and then they were subjected to flow cytometric analysis of membrane redistribution of phosphatidylserine using an annexin V and propidium iodide (PI) double-staining technique^12^.

### Confocal microscopy

Cells were seeded in glass bottom cell culture dishes (NEST, 801002) and were transfected with mRFP-GFP-tagged LC3 for 24 h. Fluorescent images of live cells were captured directly using an inverted confocal microscope (Leica). For quantification of autophagic cells, fluorescent puncta were determined from samples in triplicate^12^.

### Transmission electron microscopy

BEL7402 and HepG2 cells were fixed and embedded. Thin sections (90 nm) were examined at 80 kV with a JEOL 1200EX transmission electron microscope. Three fields containing more than 5 randomly selected microscopy-captured images were counted and autophagosomes were defined as double-membrane vacuoles measuring 0.1 or 1.0 μm.

### Animal experiments

Six week old female nude mice were subcutaneously inoculated in the right dorsal flank with BEL7402 or HepG2 cells (5 × 10^6^ cells in 100 μL PBS/mouse) to induce tumor development. When tumors reached an average volume of 200 mm^3^, tumor-bearing mice were intra-tumorally inoculated with BafA1. Mice were randomly divided into four groups (three mice per group): (a) vehicle control, (b) intraperitoneal (i.p.) treatment with JNK inhibitor (SP600125) alone, (c) intratumoral administration with BafA1, (d) BafA1 treatment in combination with SP600125, the inhibitor (SP600125) was administered one day prior to treatment with BafA1. After five weeks treatment, tumor sections (5 μm) were subjected to either hematoxylin-eosin (H&E) staining or terminal deoxynucleotidyl transferase dUTP nick end labeling (TUNEL) assay as previously described^21^.

For the *in vivo* oncolysis study, 10 mice were included in each treatment group, and each of the four groups was treated as described above for two weeks. At five day intervals, mice were examined for tumor growth or survival. Tumor diameter was measured with calipers and tumor volume was calculated based on the following formula: volume = (greatest diameter) × (smallest diameter)^2^/2. The experiment was terminated when tumors reached 1 cm^3^ in volume and/or symptomatic tumor ulceration occurred. Surviving mice were sacrificed under anesthesia. The animal experiment was conducted at Dalian Medical University (Dalian, China), complying with the national guidelines for the care and use of laboratory animal. All animal experiment were approved the experimental animal ethics committee of Dalian Medical University.

### Statistical analysis

Comparisons of data were first performed using one-way analysis of variance (ANOVA). Multiple comparisons between treatment groups and controls were evaluated using Dunnett’s least significant difference (LSD) test. For analysis of *in vivo* data, statistical significance between groups was calculated based on the LSD test using SPSS 17.0 software (SPSS Inc., Chicago, IL, USA). A p-value of p < 0.05 was considered statistically significant. All experiments were carried out in triplicate as three independent experiments.

## Additional Information

**How to cite this article**: Yan, Y. *et al.* Bafilomycin A1 induces caspase-independent cell death in hepatocellular carcinoma cells via targeting of autophagy and MAPK pathways. *Sci. Rep.* **6**, 37052; doi: 10.1038/srep37052 (2016).

**Publisher’s note:** Springer Nature remains neutral with regard to jurisdictional claims in published maps and institutional affiliations.

## Supplementary Material

Supplementary Information
