# Supplementary material for: Bafilomycin A1 induces caspase-independent cell death in hepatocellular carcinoma cells via targeting of autophagy and MAPK pathways
Source: Sci Rep. 2016 Nov 15;6:37052. doi: 10.1038/srep37052 (PMC5109251; doi:10.1038/srep37052)
Supplement: Supplementary Information [file srep37052-s1.pdf]

Bafilomycin A1 induces caspase-independent cell death in hepatocellular carcinoma cells via targeting of autophagy and MAPK pathways

Yumei Yan[a,c,1], Ke Jiang[b,1], Peng Liu[a], Xianbin Zhang[a], Xin Dong[a], Jingchun Gao[a], Quentin Liu[b], Martin P. Barr[d], Quan Zhang[e], Xiukun Hou[c,***], Songshu Meng[b,**], Peng Gong[a,*]

[a]Department of Hepatobiliary Surgery, the First Affiliated Hospital, Dalian Medical University, No. 222 Zhongshan Road, Dalian 116021, China

[b]Institute of Cancer Stem Cell, Dalian Medical University Cancer Center, 9 Lvshun Road South, Dalian 116044, China

[c]The First Department of Ultrasound, the First Affiliated Hospital to Dalian Medical University No. 222 Zhongshan Road, Dalian 116021, China

[d]Thoracic Oncology Research Group, Institute of Molecular Medicine, Trinity Centre for Health Sciences, St. James's Hospital & Trinity College, Dublin, Ireland

[e]College of Veterinary Medicine, Yangzhou University, Yangzhou, 225009, China

[1] These authors contributed equally to this work.

*Corresponding authors：

Peng Gong, Professor

Department of Hepatobiliary Surgery, the First Affiliated Hospital, Dalian Medical University,

No. 222 Zhongshan Road, Dalian, 116021, China

Tel.: 86-411-86110139

Fax: 86-411-86110139

E-mail: gongpengdalian@163.com

Songshu Meng, Ph D, Professor

Institute of Cancer Stem Cell, Dalian Medical University Cancer Center,

9 Lvshun Road South, Dalian, 116044, China

Tel/Fax: +86-411-86110496

Email: ssmeng@dmu.edu.cn

Xiukun Hou, Professor

The First Department of Ultrasound, the First Affiliated Hospital to Dalian Medical University, Dalian, 116021, China

Tel.: 86-411-83635963-3230

Fax: 86-411-83622844

E-mail: xiukunhou28@sina.com

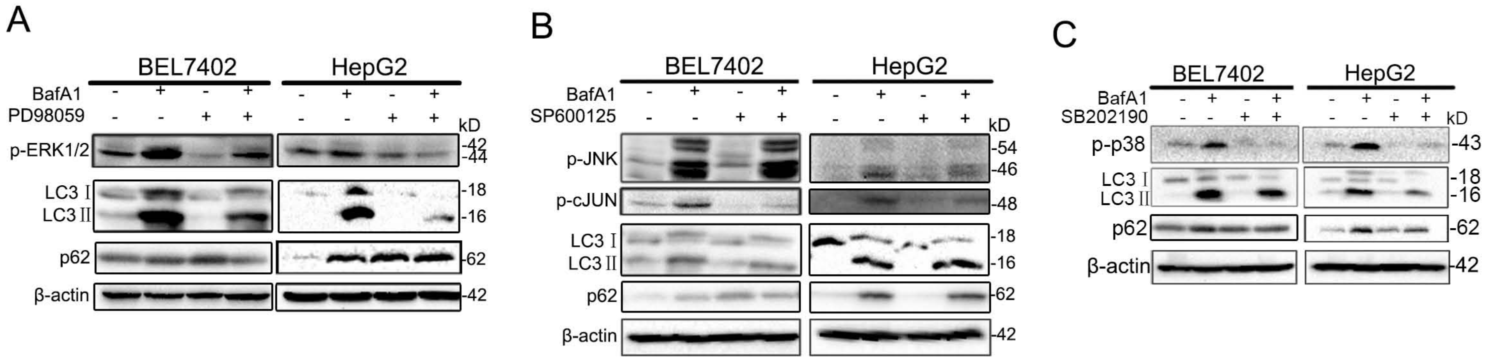

Supplementary Figure S1: Affect of MAPK inhibitors in Bafilomycin-induced autophagy (A, B, C) HCC cells were pre-treated with inhibitors to either ERK (PD98059), JNK (SP600125), or p38 (SB202190) and subsequently treated with BafA1 alone, or in combination, for 24 h. IB analysis used to examine expression of p-Erk1/2 (A), p-JNK, p-cJUN (B), p-p38 (C), LC3 and p62, using β-actin as a loading control, (BafA1 means Bafilomycin A1).

FIG2B

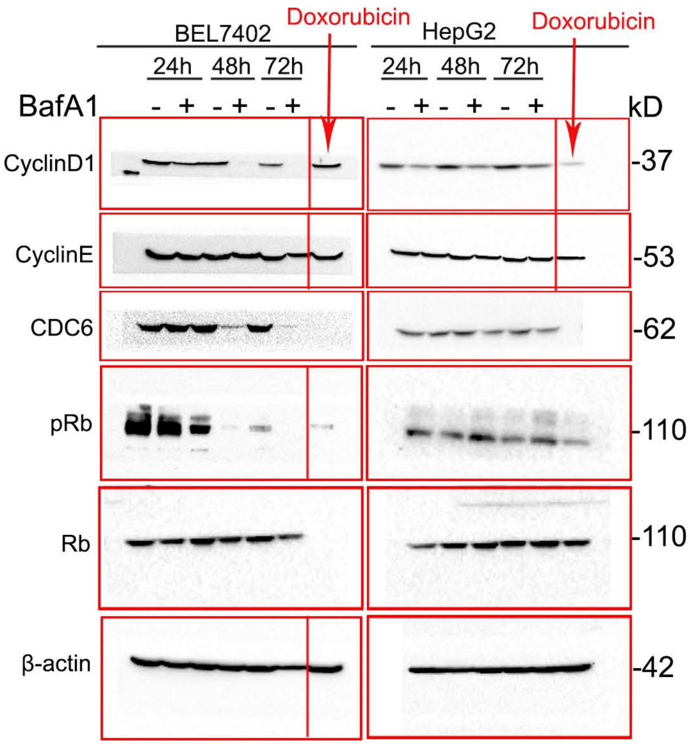

FIG2C

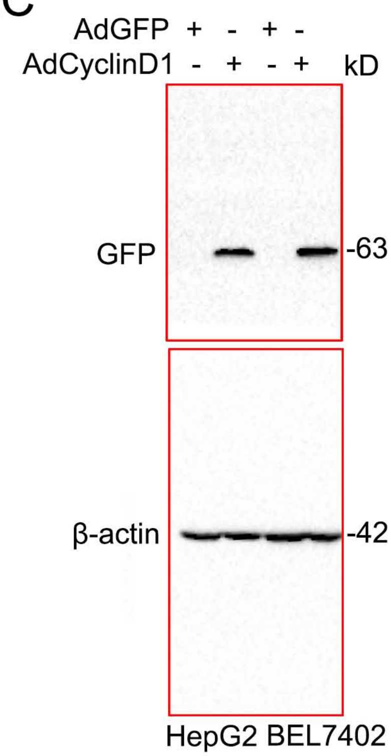

FIG2D

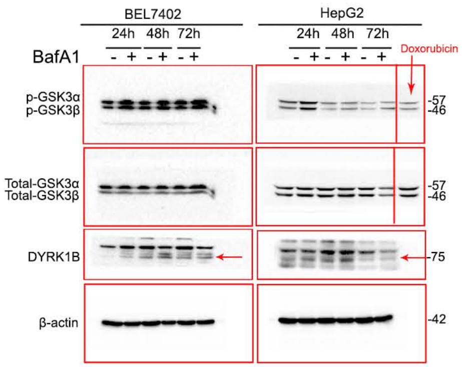

FIG2E

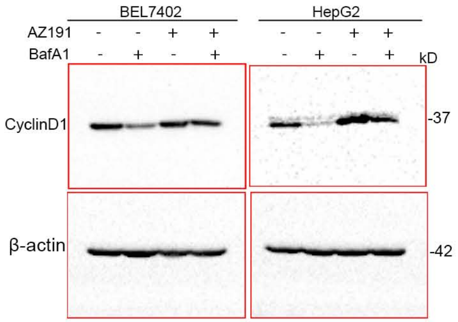

FIG2G

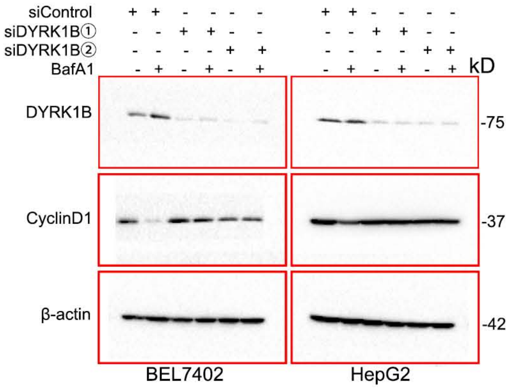

Supplementary Figure S2: Full-length images of immunoblots shown in Figure 2

FIG3B

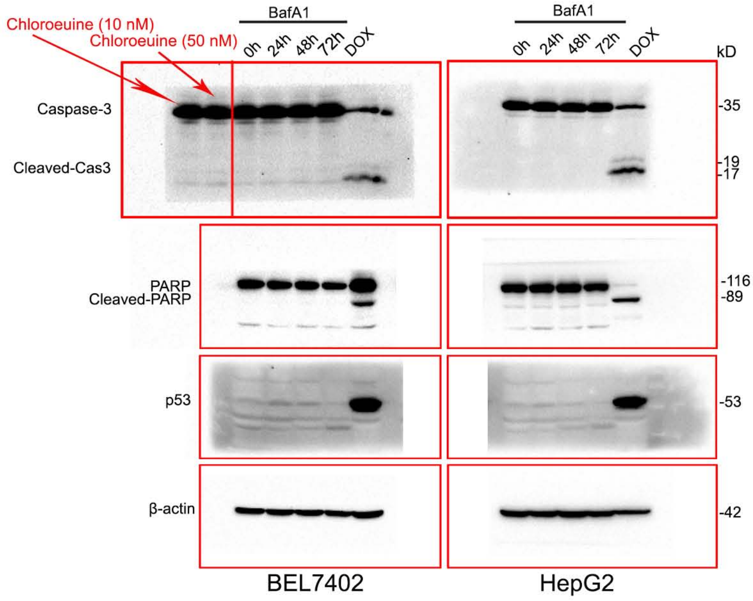

FIG3D

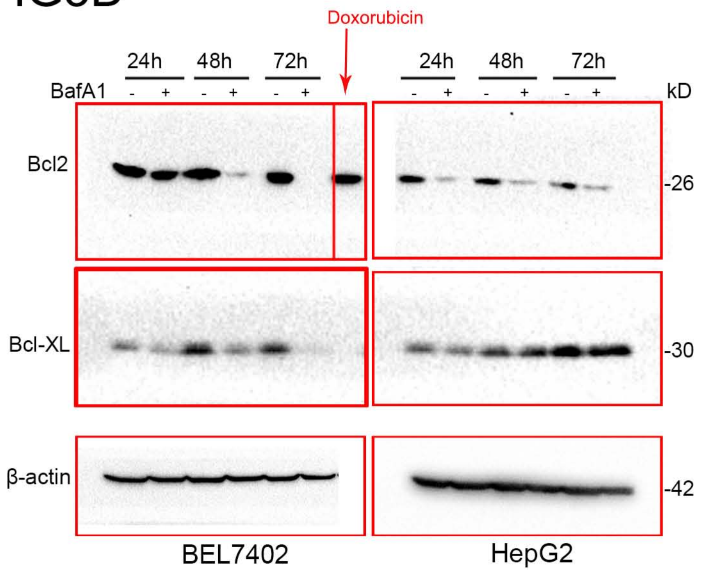

FIG3E

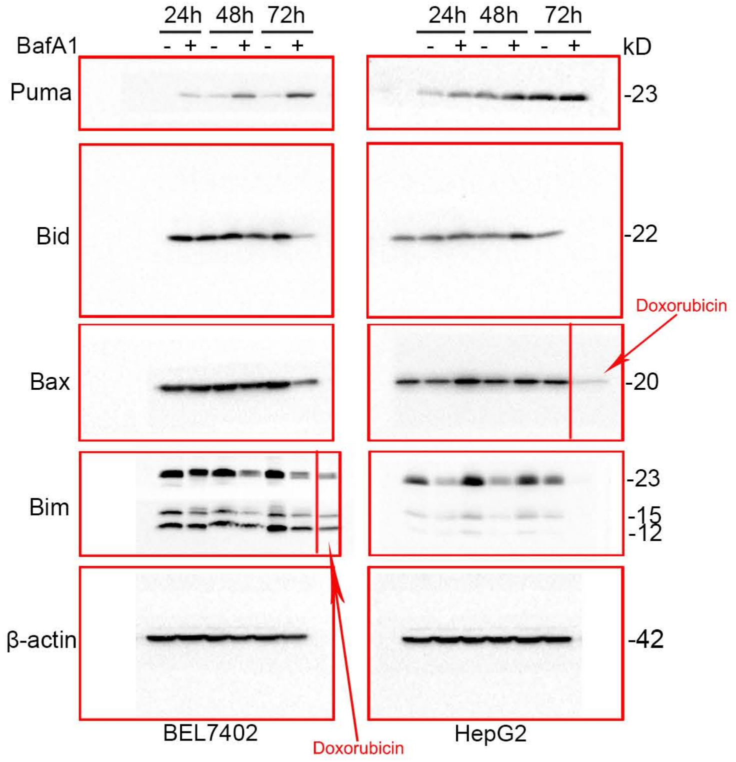

FIG3F

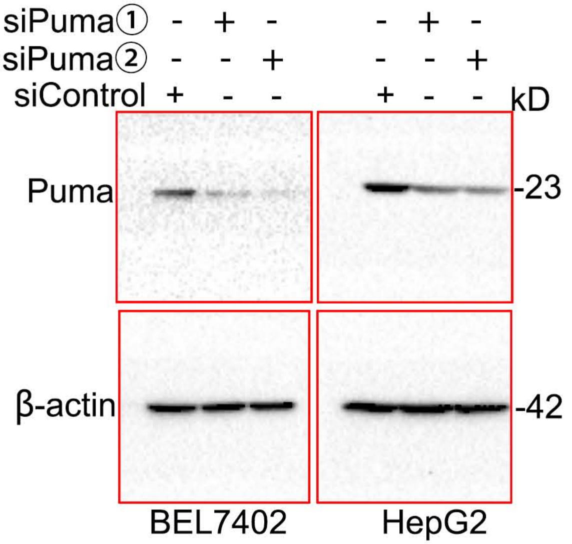

Supplementary Figure S3: Full-length images of immunoblots shown in Figure 3

FIG4B

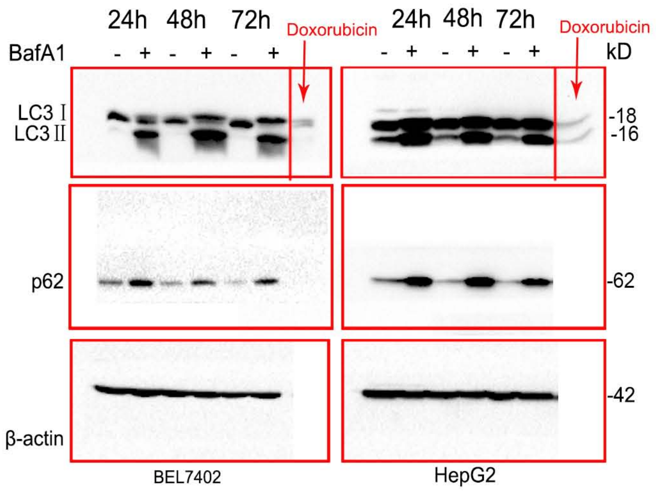

FIG5A

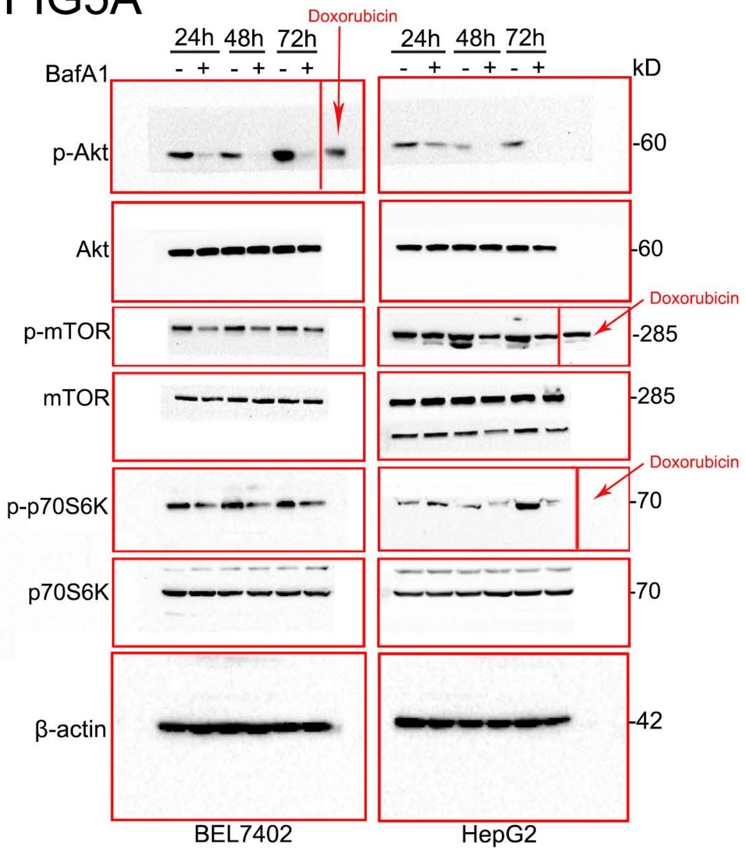

FIG5B

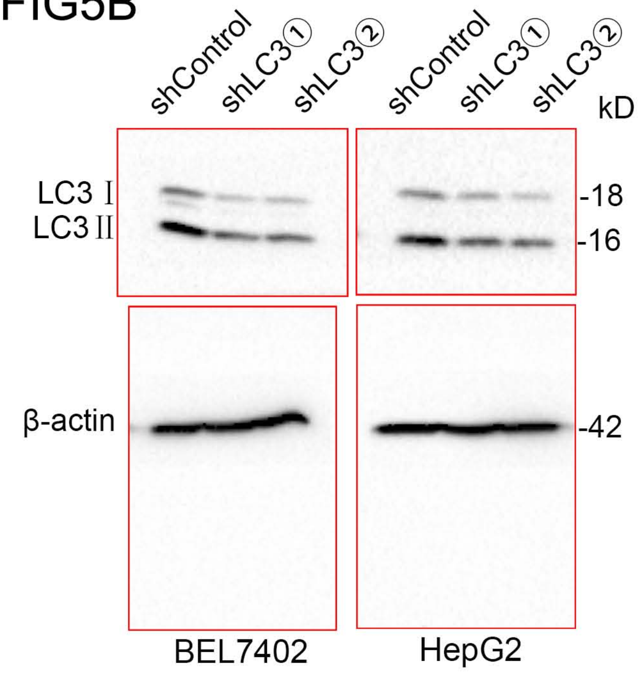

Supplementary Figure S4: Full-length images of immunoblots shown in Figure 4 and Figure 5

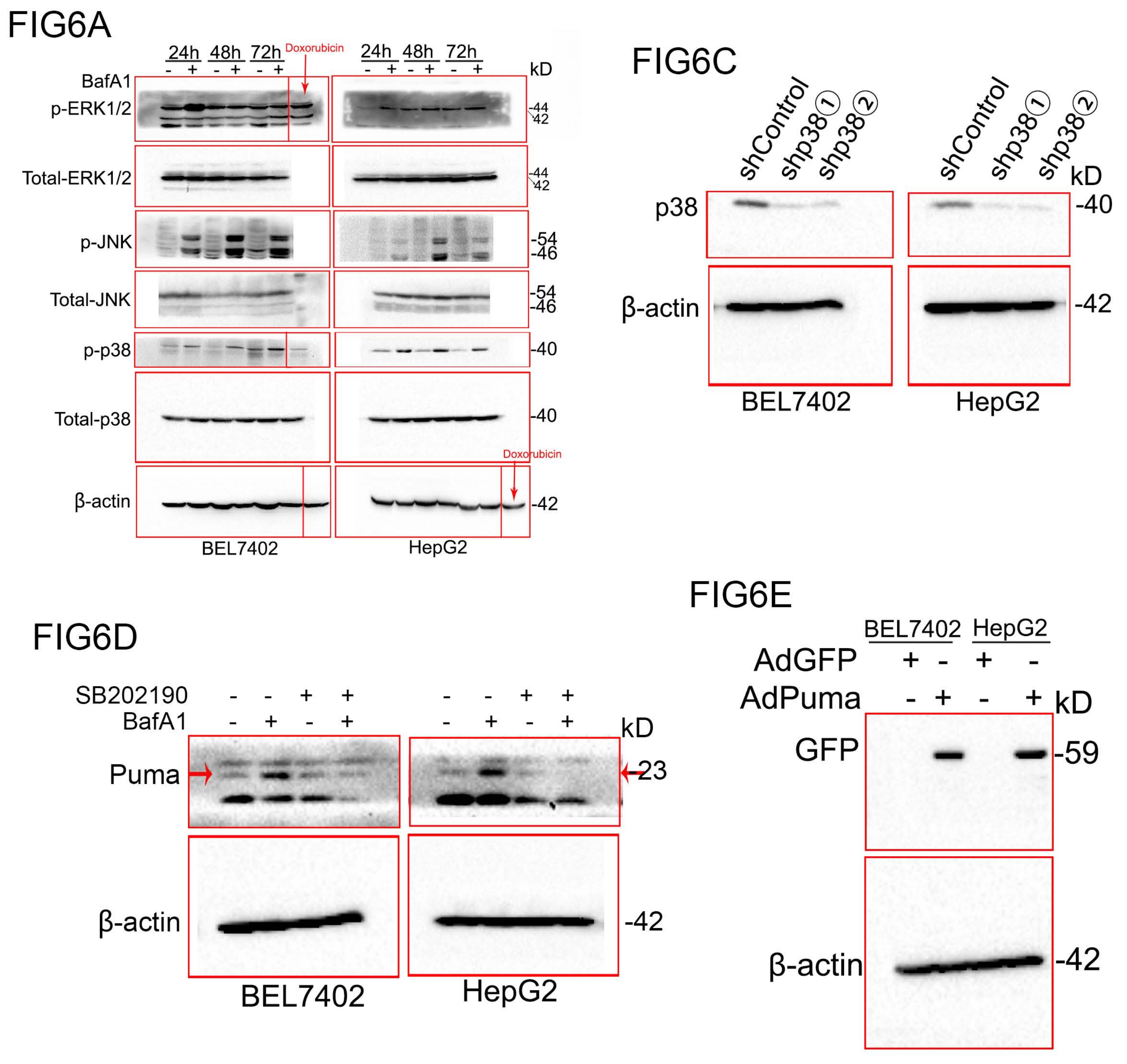

Supplementary Figure S5: Full-length images of immunoblots shown in Figure 6
